# Retraction Note: Randomized controlled trial for selective preventive transdiagnostic intervention for adolescents at risk for emotional disorders

**DOI:** 10.1186/s13034-023-00603-0

**Published:** 2023-04-28

**Authors:** Manuel Vivas-Fernandez, Luis-Joaquin Garcia-Lopez, Jose A. Piqueras, Jose-Antonio Muela-Martinez, Josefa Canals-Sans, Lourdes Espinosa-Fernandez, David Jimenez-Vazquez, Maria del Mar Diaz-Castela, Paula Morales-Hidalgo, Maria Rivera, Jill Ehrenreich-May

**Affiliations:** 1 University of Jaen, Jaen, Spain; 2 Miguel Hernandez University, Elche, Spain; 3 Universitat Rovira I Virgili, Tarragona, Spain; 4 University of Miami, Miami, USA; 5 Department of Psychology, Division of Clinical Psychology, University of Jaen, Campus de Las Lagunillas S/N, C-5, Jaen, Spain; 6 Universitat Oberta de Catalunya, Barcelona, Spain

**Retraction Note: Child and Adolescent Psychiatry and Mental Health (2023) 17:7**

10.1186/s13034-022-00550-2

The Editors-in-Chief have retracted this article because the authors have informed them that, after publication, they became aware that there were errors in some portions of the software database used for the analysis of statistical data. These errors have altered the major conclusions, including significant results presented in the text and tables. The authors have been invited to submit a new manuscript for peer review. All authors agree with this retraction.

